# Draft Genome Sequence of Cupriavidus basilensis SRS, a Bacterium Isolated from Stream Sediments

**DOI:** 10.1128/mra.00691-22

**Published:** 2022-09-08

**Authors:** He Fu, Alex Kugler, Patrick Huyck, Robin L. Brigmon, Elizabeth A. Ottesen

**Affiliations:** a Department of Microbiology, University of Georgia, Athens, Georgia, USA; b Savannah River National Laboratory, Savannah River Site, South Carolina, USA; University of Southern California

## Abstract

Cupriavidus basilensis SRS was isolated from stream sediments from the Savannah River Site in South Carolina. Here, we report the draft genome sequence and annotation of Cupriavidus basilensis SRS. The genome contains 8,918,236 bp and 7,916 predicted protein-coding genes, with a total G+C content of 65.2%.

## ANNOUNCEMENT

*Cupriavidus* species (formerly known as *Wautersia* or *Ralstonia*) are Gram-negative betaproteobacteria that are known for their diverse metabolic capabilities (1–4). In addition, heavy metal resistance is a typical characteristic of *Cupriavidus* strains. Previously sequenced *Cupriavidus* strains, such as Cupriavidus metallidurans, Cupriavidus taiwanensis, Cupriavidus basilensis, *Cupriavidus* sp. strain BIS7, and *Cupriavidus* sp. strain HMR-1, encode a number of putative proteins involved in heavy metal resistance and biodegradation activities (5–11).

Cupriavidus basilensis SRS was isolated from sediments from Tims Branch Creek (33.330008N, 81.689198W) at the Savannah River Site (Aiken, SC), which is well known to be contaminated with heavy metals and radionuclides (12–14). Briefly, sandy loam soil samples from the surface to 6 inches below the surface were collected with a stainless-steel auger and handled aseptically for subsequent analysis. The auger was cleaned and rinsed with 70% ethanol between samplings. The soil samples were transferred to sterile 50-mL centrifuge tubes (Thermo Fisher Scientific) and transported to the laboratory for immediate microbiological processing. The bacteria were grown on Difco nutrient agar (BD Biosciences, Franklin Lakes, NJ) for 3 days at 25°C. A single colony was inoculated into Reasoner’s 2A (R2A) medium (Teknova, Hollister, CA) and incubated overnight at 25°C. Then, 2 mL of the culture was centrifuged (4,000 × *g* for 5 min), and the pellet was used for DNA extraction with the Qiagen DNA blood and tissue kit. A sample of DNA (approximately 2.5 μg) was sent to GENEWIZ (South Plainfield, NJ, USA) for library preparation using the NEBNext Ultra DNA library preparation kit (New England Biolabs, Ipswich, MA) and whole-genome sequencing (2 × 150-bp paired-end sequencing) using an Illumina MiSeq instrument. A total of 25,239,038 reads were generated, with a mean quality score of 36.3. The subsequent data processing and analysis were performed in KBase (https://www.kbase.us) (15). Default parameters were used for all software unless otherwise specified. Trimmomatic (v0.36) was used to trim the raw reads (16). A total of 24,980,570 trimmed reads with a mean quality score of 36.4 were used by SPAdes (v3.15.3) for *de novo* assembly (17). The quality of the draft genome was assessed using CheckM (v1.0.18) (18).

The taxonomy of Cupriavidus basilensis SRS was classified using the Genome Taxonomy Database (GTDB) (v1.1.0) (19). FastANI (v0.1.3) was used to calculate the average nucleotide identity (ANI) (20). We found that Cupriavidus basilensis SRS shared 97.3% ANI with the type strain of Cupriavidus basilensis (DSM 11853 = CCUG 49340 = RK1) (RefSeq accession number GCF_008801925.2) (4), indicating that these two bacteria belong to the same species (21, 22).

We annotated the Cupriavidus basilensis SRS genome using the NCBI Prokaryotic Genome Annotation Pipeline (PGAP) (v6.1) (23–25). The genome contains 8,918,236 bp and 7,916 predicted protein-coding genes, with a total G+C content of 65.2% (Table 1). The genome annotation reveals numerous putative proteins responsible for metal acquisition and homeostasis, including 10 TonB-dependent siderophore transporters, 3 TonB proteins, 3 ExbB/ExbD proteins, 2 ferric iron ABC transporters, 4 magnesium and cobalt transport proteins, 8 extracytoplasmic function (ECF) sigma factors, and 7 heavy metal efflux pumps (lead-, cadmium-, zinc-, and mercury-transporting ATPases/copper-transporting P-type ATPases). The presence of these putative metal uptake- and metal efflux-related proteins in *Cupriavidus* make this organism relevant for bioremediation and bioextraction studies.

**TABLE 1:** General features of the draft genome of Cupriavidus basilensis SRS

Parameter	Finding
Size	8.92 Mb
G+C content (%)	65.2
No. of contigs	176
*N*_50_ (bp)	123,203
Coverage (×)	411
CheckM completeness (%)	99.94
CheckM contamination (%)	2.68

### Data availability.

The whole-genome sequence of Cupriavidus basilensis SRS has been deposited in DDBJ/ENA/GenBank under the accession number JAMBNM000000000. The version described in this paper is the first version. The BioProject accession number is PRJNA833476, the BioSample accession number is SAMN27996175, and the SRA accession number is SRR19544536.

## References

[1] Vandamme P, Coenye T. 2004. Taxonomy of the genus *Cupriavidus*: a tale of lost and found. Int J Syst Evol Microbiol 54:2285–2289. doi:10.1099/ijs.0.63247-0. 15545472

[2] Perez-Pantoja D, De la Iglesia R, Pieper DH, González B. 2008. Metabolic reconstruction of aromatic compounds degradation from the genome of the amazing pollutant-degrading bacterium *Cupriavidus necator* JMP134. FEMS Microbiol Rev 32:736–794. doi:10.1111/j.1574-6976.2008.00122.x. 18691224

[3] Fu H, Zhang JJ, Xu Y, Chao HJ, Zhou NY. 2017. Simultaneous biodegradation of three mononitrophenol isomers by a tailor-made microbial consortium immobilized in sequential batch reactors. Lett Appl Microbiol 64:203–209. doi:10.1111/lam.12696. 27864984

[4] Salvà-Serra F, Donoso RA, Cho KH, Yoo JA, Lee K, Yoon S-H, Piñeiro-Iglesias B, Moore ER, Pérez-Pantoja D. 2021. Complete multipartite genome sequence of the *Cupriavidus basilensis* type strain, a 2,6-dichlorophenol-degrading bacterium. Microbiol Resour Announc 10:e00134-21. doi:10.1128/MRA.00134-21. 33986075 PMC8142561

[5] Ray J, Waters RJ, Skerker JM, Kuehl JV, Price MN, Huang J, Chakraborty R, Arkin AP, Deutschbauer A. 2015. Complete genome sequence of *Cupriavidus basilensis* 4G11, isolated from the Oak Ridge Field Research Center site. Genome Announc 3:e00322-15. doi:10.1128/genomeA.00322-15. 25977418 PMC4432324

[6] Li LG, Cai L, Zhang T. 2013. Genome of *Cupriavidus* sp. HMR-1, a heavy metal-resistant bacterium. Genome Announc 1:e00202-12. doi:10.1128/genomeA.00202-12. PMC3569349 23409271

[7] Slaveykova VI, Pinheiro JP, Floriani M, Garcia M. 2013. Interactions of core–shell quantum dots with metal resistant bacterium *Cupriavidus metallidurans*: consequences for Cu and Pb removal. J Hazard Mater 261:123–129. doi:10.1016/j.jhazmat.2013.07.013. 23912077

[8] Hong KW, Thinagaran D, Gan HM, Yin WF, Chan KG. 2012. Whole-genome sequence of *Cupriavidus* sp. strain BIS7, a heavy-metal-resistant bacterium. J Bacteriol 194:6324. doi:10.1128/JB.01608-12. 23115161 PMC3486403

[9] Monsieurs P, Moors H, Van Houdt R, Janssen PJ, Janssen A, Coninx I, Mergeay M, Leys N. 2011. Heavy metal resistance in *Cupriavidus metallidurans* CH34 is governed by an intricate transcriptional network. Biometals 24:1133–1151. doi:10.1007/s10534-011-9473-y. 21706166

[10] Hajdu R, Pinheiro JP, Galceran J, Slaveykova VI. 2010. Modeling of Cd uptake and efflux kinetics in metal-resistant bacterium *Cupriavidus metallidurans*. Environ Sci Technol 44:4597–4602. doi:10.1021/es100687h. 20491434

[11] Chen W-M, Wu C-H, James EK, Chang J-S. 2008. Metal biosorption capability of *Cupriavidus taiwanensis* and its effects on heavy metal removal by nodulated *Mimosa pudica*. J Hazard Mater 151:364–371. doi:10.1016/j.jhazmat.2007.05.082. 17624667

[12] Thomas JC, Oladeinde A, Kieran TJ, Finger JW, Bayona-Vásquez NJ, Cartee JC, Beasley JC, Seaman JC, McArthur JV, Rhodes OE, Glenn TC, Jr. 2020. Co‐occurrence of antibiotic, biocide, and heavy metal resistance genes in bacteria from metal and radionuclide contaminated soils at the Savannah River Site. Microb Biotechnol 13:1179–1200. doi:10.1111/1751-7915.13578. 32363769 PMC7264878

[13] Evans A, Bauer L, Haselow J, Hayes D, Martin H, McDowell W, Pickett J. 1992. Uranium in the Savannah River Site environment. Westinghouse Savannah River Co., Aiken, SC.

[14] Kaplan DI, Smith R, Parker CJ, Baker M, Cabrera T, Ferguson BO, Kemner KM, Laird M, Logan C, Lott J, Manglass L, Martinez NE, Montgomery D, Seaman JC, Shapiro M, Powell BA. 2020. Uranium attenuated by a wetland 50 years after release into a stream. ACS Earth Space Chem 4:1360–1366. doi:10.1021/acsearthspacechem.0c00124.

[15] Arkin AP, Cottingham RW, Henry CS, Harris NL, Stevens RL, Maslov S, Dehal P, Ware D, Perez F, Canon S, Sneddon MW, Henderson ML, Riehl WJ, Murphy-Olson D, Chan SY, Kamimura RT, Kumari S, Drake MM, Brettin TS, Glass EM, Chivian D, Gunter D, Weston DJ, Allen BH, Baumohl J, Best AA, Bowen B, Brenner SE, Bun CC, Chandonia J-M, Chia J-M, Colasanti R, Conrad N, Davis JJ, Davison BH, DeJongh M, Devoid S, Dietrich E, Dubchak I, Edirisinghe JN, Fang G, Faria JP, Frybarger PM, Gerlach W, Gerstein M, Greiner A, Gurtowski J, Haun HL, He F, Jain R, et al. 2018. KBase: the United States Department of Energy Systems Biology Knowledgebase. Nat Biotechnol 36:566–569. doi:10.1038/nbt.4163. 29979655 PMC6870991

[16] Bolger AM, Lohse M, Usadel B. 2014. Trimmomatic: a flexible trimmer for Illumina sequence data. Bioinformatics 30:2114–2120. doi:10.1093/bioinformatics/btu170. 24695404 PMC4103590

[17] Bankevich A, Nurk S, Antipov D, Gurevich AA, Dvorkin M, Kulikov AS, Lesin VM, Nikolenko SI, Pham S, Prjibelski AD, Pyshkin AV, Sirotkin AV, Vyahhi N, Tesler G, Alekseyev MA, Pevzner PA. 2012. SPAdes: a new genome assembly algorithm and its applications to single-cell sequencing. J Comput Biol 19:455–477. doi:10.1089/cmb.2012.0021. 22506599 PMC3342519

[18] Parks DH, Imelfort M, Skennerton CT, Hugenholtz P, Tyson GW. 2015. CheckM: assessing the quality of microbial genomes recovered from isolates, single cells, and metagenomes. Genome Res 25:1043–1055. doi:10.1101/gr.186072.114. 25977477 PMC4484387

[19] Chaumeil PA, Mussig AJ, Hugenholtz P, Parks DH. 2019. GTDB-Tk: a toolkit to classify genomes with the Genome Taxonomy Database. Bioinformatics 36:1925–1927. doi:10.1093/bioinformatics/btz848. PMC7703759 31730192

[20] Jain C, Rodriguez-R LM, Phillippy AM, Konstantinidis KT, Aluru S. 2018. High throughput ANI analysis of 90K prokaryotic genomes reveals clear species boundaries. Nat Commun 9:5114. doi:10.1038/s41467-018-07641-9. 30504855 PMC6269478

[21] Figueras MJ, Beaz-Hidalgo R, Hossain MJ, Liles MR. 2014. Taxonomic affiliation of new genomes should be verified using average nucleotide identity and multilocus phylogenetic analysis. Genome Announc 2:e00927-14. doi:10.1128/genomeA.00927-14. 25477398 PMC4256179

[22] Richter M, Rossello-Mora R. 2009. Shifting the genomic gold standard for the prokaryotic species definition. Proc Natl Acad Sci USA 106:19126–19131. doi:10.1073/pnas.0906412106. 19855009 PMC2776425

[23] Li W, O'Neill KR, Haft DH, DiCuccio M, Chetvernin V, Badretdin A, Coulouris G, Chitsaz F, Derbyshire MK, Durkin AS, Gonzales NR, Gwadz M, Lanczycki CJ, Song JS, Thanki N, Wang J, Yamashita RA, Yang M, Zheng C, Marchler-Bauer A, Thibaud-Nissen F. 2021. RefSeq: expanding the Prokaryotic Genome Annotation Pipeline reach with protein family model curation. Nucleic Acids Res 49:D1020–D1028. doi:10.1093/nar/gkaa1105. 33270901 PMC7779008

[24] Haft DH, DiCuccio M, Badretdin A, Brover V, Chetvernin V, O'Neill K, Li W, Chitsaz F, Derbyshire MK, Gonzales NR, Gwadz M, Lu F, Marchler GH, Song JS, Thanki N, Yamashita RA, Zheng C, Thibaud-Nissen F, Geer LY, Marchler-Bauer A, Pruitt KD. 2018. RefSeq: an update on prokaryotic genome annotation and curation. Nucleic Acids Res 46:D851–D860. doi:10.1093/nar/gkx1068. 29112715 PMC5753331

[25] Tatusova T, DiCuccio M, Badretdin A, Chetvernin V, Nawrocki EP, Zaslavsky L, Lomsadze A, Pruitt KD, Borodovsky M, Ostell J. 2016. NCBI Prokaryotic Genome Annotation Pipeline. Nucleic Acids Res 44:6614–6624. doi:10.1093/nar/gkw569. 27342282 PMC5001611

